# Water Conductors and the Artesian Well at Charleston, S. C.

**Published:** 1880-03

**Authors:** Middleton Michel

**Affiliations:** Chairman of Committee on Epidemics and Public Hygiene, Charleston, S. C.; No. 39 Society Street


ARTICLE II.

### WATER CONDUCTORS AND THE ARTESIAN WELL AT CHARLESTON, S. C.

REPORT OF PROFESSOR MIDDLETON MICHEL, M. D.
(Chairman of Committee on Epidemics and Public Hygiene, Charleston, S. C.)

GENTLEMEN:—After some delay in reply to the call of Council upon this Board to determine the question of the possible contamination of the water supply about to be furnished the city of Charleston, your committee consider themselves prepared to offer a full and determinate report.

It is proper to observe that responsible caution governs the expression of opinion relating to so important a subject as the purity of the water we are to drink, and that a thorough investigation of every part of the inquiry, irrespective of the time or labor it involved, has been a duty with us as custodians of the public health, the discharge of which is the more imperative since a general impression—the most fallacious of all evidence—that this water must of necessity become polluted, has greatly exercised the thinking public.

An array of prerogative facts are the only kind, it must be remembered, which satisfy the demands of science in an investigation of this sort, and on such alone, therefore, shall we rely.

In this, as on many other occasions, we see a signal illustration of the truth that popular anxieties are often found drifting into the misdirected channels of true danger. There are those who are ready to declare that they would not exchange our accustomed cistern water for artesian water delivered to them through iron pipes by pressure, in a constant flow and with fixed velocity, though a sanitarian knows that brick cemented yet porous walled cisterns cannot be depended upon as being water-tight. It is therefore strange that we should hear complaints about the permanent purity of water rushing onward through tested iron pipes, under a pressure of fifty pounds to the square inch, from those who have never raised an objection to the purity of our cistern water, imperfectly protected from soil infiltration and in stagnant repose, within a porous vault already surcharged with organic detritus washed down into it from our roofs. In a recent sanitary survey of Memphis, on the eve of being presented to the National Board of Health, Dr. R. W. Mitchell, a member of that Board, finds the water of five hundred and thirty cisterns thus far analyzed so polluted that an immediate interdict on the use of one-third of them appears called for. This befouling of cisterns, readily ascertained by the chlorine determinations in the analysis, has rarely if ever been evoked to account for the origin of yellow fever, typhoid, or any other disease, and is only here referred to in order to show that such pollution exists. We intentionally exclude from our report any discussion of the "drinking-water theory," the debatable problem of the filth-origin of disease (pythogenetic), and will not enter upon that line of argument which discusses disease as originating *within* the patient so that he infects himself (autogenetic) or from one patient to another (heterogenetic.) We shall not describe the particular effects of tainted food, whether solid or fluid, upon the body, how impure water may be drank, or what becomes of the epicure who never eats his game until it is crawling off the table. But in pretermitting this discussion, properly the province of the medical scientist, we shall confine ourselves, in the direction which the solution of our problem must take, to the other department of sanitary science that engages the services of the sanitary engineer. In conveying potable water in conduits and its distribution by mains and service-pipes there are questions of hygiene involved, as when lead-pipes are used, with which an aptly appointed scientist is presumed to be acquainted. Thus, the best material to be employed is first recognized and suggested by the medical sanitarian; the proper consummation of the work, however, belongs to the engineer. Now, the material employed for the main distribution pipes has been determined, and should be of *cast*, not wrought iron, subjected to a particular process in the method of moulding, pouring, coating and hydraulic proof. Have these conditions been complied with in the case before us? Your committee ascertains that they have. The pipe *moulds* are from the foundry of R. D. Wood & Co. and appear to have secured all accuracy of form and thickness, through a careful tempering of the sand and drying of the mould so to withstand the trying action of the molten metal. In *pouring* the castings are protected from sudden chills while cooling. To protect the pipes from corrosion the process of *coating* is then had recourse to, which we find is the one devised by Dr. R. Agues Smith and universally approved of. This process is as follows: The pipes, free from rust, are heated to a temperature of 500 degrees Fahrenheit, until the pores of the iron are well opened, they are then dipped perpendicularly into a hot bath of coal-tar varnish, previously deodorized, the naphtha removed, and here remain until a varnish coating has perfectly formed on both faces of the casting. The *hydraulic* testing machine, which we shall not describe, though the drawing is before us, next subjects each pipe to a pressure test of three hundred pounds per square inch, and if evidences of porousness or weakness are found the pipe is condemned. In this connection the *durability* or life of the pipe, as it is called, is important. We have ascertained that its minimum duration is thirteen or seventeen years, its maximum perhaps over one hundred years. The Hon. A. W. Craven, formerly chief engineer of the Croton Aqueduct Department, New York, says: " In cast iron you are dealing with a certainty. Pipe which has been in constant use for one hundred years, and unprotected by any coating, either on its interior or exterior surfaces, has been examined, and to no appreciable extent was there any dimunition in its weight or strength. It is the opinion of those engineers who have had most experience in and given most study to this subject, that we have not had an opportunity to define the limit to the duration of cast-iron pipe used for aqueduct purposes." From a sanitary standpoint, then, such a material and such a surface is practically as good a one as could be desired or obtained, and of such a nature your committee find the pipes to be that are being laid in this city.

The mode of junction of these pipes is not without its bearing upon the water they transmit, and must here be considered. The method usually adopted and described in our works on hygiene is the one here carried out. The hub and spigot joint is packed with tow, and then melted lead is run in and driven up with a set. Now, we must state that the tow may impart for a while an unpleasant taste to the water; for in Europe this was found to be the case, and it has, therefore, been recommended that the joints should be carefully painted with Portland cement. This has not been done; but, from the nature of the operations which we have inspected, we do not think this will affect the water deleteriously, and, from the relative expansive power of lead and iron, the former being two and a half times greater than the latter, the joint only becomes the tighter.

In the engineering of these practically impermeable iron-pipes there seems necessary that two chief indications should be fulfilled. These are: Their location at a certain depth, controlled in each latitude by terrine conditions, and the observance of a strict level course; that by the first condition they shall be protected from all injury of traffic or frost, and by the second that they may deliver their contents without impediment. In the maintenance of these rules we must at once perceive what diversified relations water-pipes must bear to drains, sewers, culverts and like structures in different parts of the world. Thus in one city whose drainage is perfect there may be no communication with drain or sewer; in another less favored locality the pipes in a few places may pass directly *through sewers*, as in Louisville, Ky., while in Paris a double set of sewers exist, and in one set, carrying the ordinary drainage, water-pipes pass *through* their whole length.

It is just here that we should inquire, what would be the nature of this engineering work done in a city like our own; in what way would it have to be performed, and what would the sanitary significance of the work when accomplished? That the engineer must observe his fixed rules here as elsewhere is obvious, but what relation will his work bear to the drainage arrangement of Charleston? We speak advisedly of simply a drainage arrangment, since actually no system in the matter obtains. Unfortunate as this may be, how much greater would have been this evil if it actually related to sewage matter, properly speaking to excremental defilement, which happily is not the case. Upon this point we cannot too stringently insist, as this has everything to do with the veritable question of the propagation of disease through the dejecta and emanations from the patient himself. To entirely appreciate our meaning, then, we must recall the flat site of our seaside town situated between two rivers and largely saturated, always percolated, by salt water; there must be a free circulation of salt water through the pores of our soil, when we consider the diurnal rise and fall of the tide; whatever we may say of the moisture of the soil which ensues, we can affirm at least that stagnation of water and changes in the soil are thereby largely prevented. To remedy this hygrometric condition when carried to excess, attempts at drainage independently undertaken have resulted in the course of time, as might have been expected, in as incongruous and inefficient a piece of work as could be exhibited in any part of the wide world; but, however imperfect, as we have already said, it does not involve the question of sewage proper, excrementitious matter *per se*, as is meant in all other cities in which we speak of sewers and sewerage. There are drains in our city not sewers, for soil moisture, not animal or human excreta, receptacles of surfacial deposits, and from deeper layers of salt water, itself an antiseptic. To return to the subject, then, these imperfect laterals, of irregular construction, built of different materials, running into the mains at different heights, constitute an obstacle to the passage of water-pipe at any depth; the natural consequence of which has been that maintaining the required level the pipe passes sometimes above, sometimes below, and in some *few* instances—ascertained, numbered, and now before your committee—transfixes or *intersects* the drain itself; but even where this has occurred it must not be forgotten that no pipe rests in sewage matter in Charleston, since these drains contain the foul soil moisture and domestic wastes of kitchen and yard, upon much of which, did time and space permit, we could show the wonderful septic effect of the earth through which percolation has taken place. We dwell pertinently on the fact that no drain is filled with human excreta, hence this circumstance is connected with the early history of the old primitive Charlestown without a water supply, when it was not willing to incur the expense necessary to secure what many would term, perhaps, the benefits of the sewerage system as it exists elsewhere; resort was had therefore to vaults more or less remote from the dwelling-house, a primitive state of things which obtains throughout this city to this day, and which under the modifications of structure and with annual removals of their contents by the pneumatic odorless excavator, as proposed and about to be realized by this Board, inaugurates a safer system of disposing of and utilizing sewage than where water-supply and sewage are combined. At one stage of our inquiries on this point we were led to suspect that three particular drains connected with the Roper Hospital in Mazyck street might possibly form an exception to this general rule, and as there was doubt expressed your committee requested the city engineer to reopen these for our careful inspection. This was done, and it was ascertained that the water-pipe passed four feet above the lateral, which itself enters the main at seven feet beneath the surface; and again it was further ascertained that this drain, the principal hospital drain, no longer communicated, as it once formerly did, with the vaults, now dry, depots of this institution; while the other two drains only served to collect the surface moisture from the enclosure and garden in front of this building. Again, in two other instances under our observation, in which the yard drains were obscurely connected with the street laterals, these drains were re-established and made to connect with the mains through six-inch iron pipes, securing for these two residences in King and in George streets the most perfect and approved method of house and lot drainage known. Along Rutledge to Calhoun street, where an excess of salt water might be supposed to exercise some influence over the durability of the iron, it should be stated that no probable bad results need be apprehended, since on this very subject, the effects of salt water upon iron piping, experiments have been made at Cherbourg, on the coast of France, which proved perfectly satisfactory to the engineering corps.

In presence of such facts as have been elicited by this research, we believe ourselves authorized in offering a perfectly unbiased opinion on the proper subject of this report, one which indeed much concerns each and every one of us in the future. Considering the impermeability of these cast-iron pipes, their inside strength and known durability, the constant delivery system, which is the contract, the true nature of the contents of our drains, and the head pressure of fifty pounds to the square inch, there seems to us no theoretical probability even of omosis taking place, no gaseous penetration appears possible with the rush of water outward in the event of actual clearage or perforation, and even should an oblique intercommunication between drain and pipe obtain, instituting what is called negative pressure, the suction exerted on the hydraulic theory of Venturi, known to all physicists, could reach no practically appreciable extent under the conditions of the problem before us. On the contrary, gaseous emanations would sooner percolate imperfectly constructed drains of wood and clay, brick and mortar, and reaching the porous earth would be decomposed and rendered inert. Should sewage leak along the sides of the water-pipe to the nearest joint in reach, constructed as are these joints, insoluble lead compounds at most would be formed.

But, admitting the pipes laid actually in sewage, or in close proximity with nitrogenous substances, undergoing retrogressive metamorphoses, resolving themselves into elementary compounds, we have now to mention an all-important fact of chemical import which appears to have escaped all public notice, and which, nevertheless, clinches the argument and settles the question: Iron water-pipes have a distinct chemical value. Professor Medlock proved by analysis, several years ago, that iron, by its action on nitrogenous organic matter, produces nitrous acid, which Muspratt called "Nature's Scavenger." The distinguished chemist last mentioned declares that when water has remained in long contact with a large surface of iron every trace of organic matter was in two days rendered insoluble or wholly destroyed. Medlock confirmed the same results in his examination of the Amsterdam water which presented a bad taste and smell. He analyzed water brought in contact with iron, and water which had not touched iron, with the result that this latter contained 2.10 grains of organic matter, and 0.96 grain iron; the other gave but a trace of both, the organic matter was plainly shown to be either decomposed or thrown down by contact with iron, and this water was found when filtered to be clear, inodorous, of good taste, and free from organic matter.

We conclude our remarks by stating that no apprehension need be entertained, in our judgment, of the purity of our promised water supply in future, which will doubtless reach us less defiled than that which our cisterns have so long furnished, while we would recommend that, in this city, as in all other cities provided with water works of the kind, a semi-annual examination and analysis of the water be made the subject of an official report, by some one properly qualified and appointed to perform this duty; and we would also remark that, should there be any obstruction to the drains in any special locality likely to interfere with soil drainage of any lot in this city, in the judgment of this Board, they hold themselves prepared, upon being notified of the same, to see that the difficulty be rectified.

No. 39 Society Street.

